# Collagen IV levels are elevated in the serum of patients with primary breast cancer compared to healthy volunteers

**DOI:** 10.1038/sj.bjc.6604443

**Published:** 2008-06-17

**Authors:** C Mazouni, B Arun, F André, M Ayers, S Krishnamurthy, B Wang, G N Hortobagyi, A U Buzdar, L Pusztai

**Affiliations:** 1 Laboratoire de transfert biologique et oncologique, Marseille University, Houston, TX, USA; 2 Department of Breast Medical Oncology, The University of Texas MD Anderson Cancer Center, Houston, TX, USA; 3 Department of Pathology, The University of Texas MD Anderson Cancer Center, Houston, TX, USA; 4 Institut Goustave Roussy, Villejuif, France; 5 Bristol Myers Squibb Co, Princeton, NJ, USA

**Keywords:** serum collagen IV, breast cancer, primary chemotherapy, collagen IV, HER-2

## Abstract

Collagen IV is a major component of the vascular basement membrane and may be a marker of angiogenesis. Serum levels of this protein are elevated in some human cancers. Our objectives were to compare collagen IV levels in the serum of breast cancer patients and healthy women and to examine changes during preoperative chemotherapy. Sera from 51 patients with stage II–III breast cancer and 55 healthy controls were analysed. Collagen IV level was measured by a commercially available sandwich enzyme link immunoassay. Baseline serum levels were compared between cancer patients and healthy women and paired pre- and post-chemotherapy measurements were also performed in 39 patients who received preoperative chemotherapy and were correlated with response to therapy. The median serum collagen IV concentration was significantly higher in cancer patients (166 μg l^−1^) than in healthy women (115 μg l^−1^), *P*<0.001. Chemotherapy induced a significant further increase in serum collagen IV (167 μg l^−1^ prechemo *vs* 206 μg l^−1^ postchemo, *P*=0.001). There were no correlations between baseline collagen IV levels and response to therapy, age, clinical stage or HER2 status. In conclusion, patients with breast cancer have elevated levels of collagen IV compared to healthy women and collagen IV levels increase further during chemotherapy.

Collagen IV is a major component of the basement membrane that surrounds tumour vasculature (Yurchenko and O'Rear, 1993; Aumailley and Gayraud, 1998; Baluk *et al*, 2003). Collagen IV is synthesized during angiogenesis and is deposited around the endothelial cells and pericytes and could be considered as a marker of angiogenic activity (Baluk *et al*, 2003). Elevated collagen IV levels were also documented in patients with liver cirrhosis and chronic renal diseases compared to healthy individuals (Hong *et al*, 1995; Xu *et al*, 2002). Degradation of this protein occurs during invasion and metastatic spread of cancer (Robledo *et al*, 2005). High collagen IV levels were detected in the serum of patients with metastatic breast, colorectal, gastric and lung cancers as well as primary liver cancer (Ambiru *et al*, 1995; Hong *et al*, 1995). One abstract reported that baseline levels of this protein and changes during treatment may be markers of response to antiangiogenic therapy in cancer (Ayers *et al*, 2007). Collagen IV mRNA is highly expressed in breast cancer stroma and immunohistochemistry studies indicated that this protein was a major component of breast tumour vasculature (Lipponen *et al*, 1994; Symmans *et al*, 2003). However, serum collagen IV levels were not previously measured in breast cancer. We hypothesized that serum collagen IV levels maybe elevated in patients with primary breast cancer relative to healthy women because of increased angiogenic activity in the cancer. The goal of this study was to test this hypothesis and also to assess changes in collagen IV levels in a subset of patients during preoperative chemotherapy.

## Methods

### Patients and samples

Archived serum specimens were used for this research. Sera were obtained at baseline (before therapy) and after completion of 6 months of preoperative chemotherapy but before surgery from 39 HER-2-positive breast cancer patients who participated in a preoperative clinical trial. Patients were randomised to receive either four cycles of paclitaxel followed by four cycles of fluorouracil, cyclophosphamide and epirubicin or the same chemotherapy in combination with trastuzumab. Results of this clinical trial were reported previously (Buzdar *et al*, 2005). Aliquots of the same serum samples were previously used to measure changes in serum troponin levels and in circulating HER-2 receptor domain during preoperative therapy (Buzdar *et al*, 2005; Mazouni *et al*, 2007). We requested additional serum specimens from an investigator-initiated serum bank (Dr B Arun) that included sera from 12 more cancer patients with HER2-normal (*n*=8), HER-2-positive (*n*=1) or HER-2 unknown disease (*n*=3) and from 55 healthy women volunteers whose serum was accrued during the same time period as those of the cancer patients. Volunteers with history of liver disease or other chronic inflammatory condition were excluded. All cancer patients were free of liver metastasis or any acute or chronic liver or kidney disease. The volunteers were slightly younger than the cancer patients; the median ages were 46 years (range, 21–70 years) and 52 years (range, 22–68 years) respectively, (*P*=0.04). Table 1 summarises clinical characteristics of the cancer patients. Only two patients had a recurrence in the entire cancer cohort due to short median follow up.

The total number of cases included in this study is 107 (51 cancer and 55 healthy women). The total number of specimens is 146 including 107 baseline measurements and 39 repeat measurements before and after completion of 6 months of chemotherapy in a subset of the cancer patients who participated in the preoperative clinical trial. All samples were centrifuged and aliquoted after collection and stored at −80°C until the assays were performed. No repeat freezing or thawing was permitted. All patients and volunteers signed informed consent before collection of serum or tumour specimens at the UT MD Anderson Cancer Center and the local Medical Ethics Committee (IRB) approved this laboratory study on stored specimens.

### Collagen IV immunoassay

Serum collagen IV was measured with a commercially available enzyme immunoassay using monoclonal antibodies against the central triple helical region of human type IV Collagen (Collagen IV ELISA, Biotrin, Daiichi Fine Chemical Co. Ltd., Ireland). The measurements were performed following the instructions of the manufacturer. The coefficients of variation for both inter- and intra-assay variability of the assay were previously reported and were ⩽4.0% (Obata *et al*, 1989). A standard dilution of collagen IV supplied with the assay was used to calibrate the measurements.

Six formaldehyde-fixed paraffin-embedded primary tumour specimens that matched baseline serum samples could also be retrieved from the institutional tumour bank. Immunohistochemical staining was performed after antigen heat retrieval in citrate buffer (pH=6) in a steamer for 10 min. Primary antibodies were used against CD34 (BD Bioscience, 1 : 20 dilution) and VEGF (Santa Cruz, 1 : 10). A standard three-step avidin–biotin peroxidase complex technique was used for visualisation of the antigens using DAB as the chromogen. A distinct brown staining in the cytoplasm of the cells was regarded as positive staining.

### Statistical analysis

*χ*^2^ or Fisher exact tests were used to compare differences between categorical variables. Differences in continuous variables were analysed using the Mann–Whitney *U*-tests. The Wilcoxson test was used to compare collagen IV concentration before and after treatment. A *P*-value <0.05 was considered significant. The correlation between age and serum collagen IV levels was assessed using the Spearman's correlation coefficient. Statistical analyses were performed with SPSS® software version 12.0.

## Results

### Baseline serum collagen IV levels differ between breast cancer patients and healthy women

**Figure 1:**
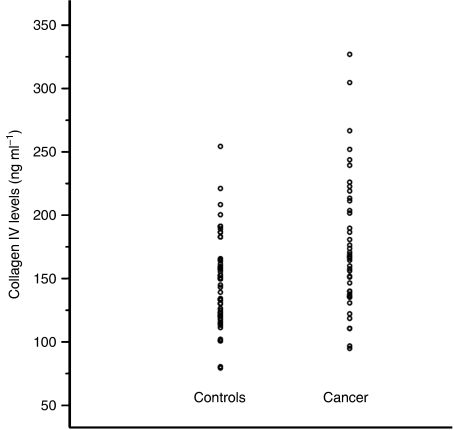
Comparison of median concentration of collagen IV between breast cancer patients (*n*=51) and healthy volunteers (*n*=55).

The median serum collagen IV concentration was 166 ng ml^−1^ (range, 85–327 ng ml^−1^) in breast cancer patients (*n*=51) and it was 115 ng ml^−1^ (range, 61–339 ng ml^−1^) in healthy volunteers (*n*=55) (Figure 1). Mean serum levels were also different 174 *vs* 118 ng ml^−1^, respectively and these differences were statistically significant (*P*<0.001). However, individual measurements overlapped substantially between the two groups. Next, we correlated collagen IV levels with clinical stage. The median concentration was 178 ng ml^−1^ in stage I cancers (*n*=10), it was 156 ng ml^−1^ in stage II (*n*=29), and 167 ng ml^−1^ in stage III (*n*=10) tumours. Clinical stage could not be determined for two cases (*P*=0.21). These differences were not significantly different, *P*=0.21. These results indicate that collagen IV concentrations are generally higher in the serum of women with breast cancer compared to healthy individuals, but individual variations are substantial and are not related to clinical stage.

We also examined the influence of age on collagen IV levels, mean (139.1 *vs* 151.2 ng ml^−1^, *P*=0.25) and median (122.6 *vs* 137.8 ng ml^−1^, *P*=0.06) collagen IV levels were not significantly different between women younger than 50 years of age (*n*=56) or older (*n*=50) years of age with or without cancer. There was no relationship between age and collagen IV levels (*r*=−0.102, *P*=0.48). We performed an exploratory analysis and compared collagen IV levels between HER2-positive (*n*=40) and HER-2 normal cancers (*n*=8) at baseline, there was no significant difference in the mean (166.3 *vs* 176 ng ml^−1^, *P*=0.61) or median (156.9 *vs* 169.2 ng ml^−1^, *P*=0.29) collagen IV levels between these 2 groups.

Unfortunately, we could not retrieve most of the primary tumour biopsies from the patients who were included in the serum study to correlate angiogenic activity within the primary tumour and serum collagen IV levels. Immunohistochemistry results for collagen IV (CD34) and VEGF were available on six cases. There was no clear correlation between IHC staining for these markers and collagen IV levels.

### Changes in collagen IV levels after preoperative chemotherapy

**Figure 2:**
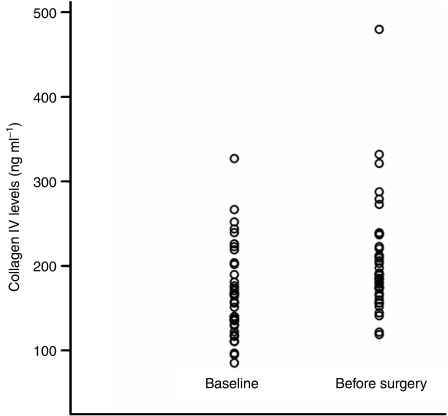
Median serum collagen IV concentrations at baseline and after completion of 6 months of preoperative chemotherapy (*n*=39).

The mean serum collagen IV concentration prior to chemotherapy was 167 ng ml^−1^ (range, 85–327) in the 39 patients who subsequently received 6 months of preoperative chemotherapy with or without trastuzumab. The mean serum collagen concentration had risen to 206 ng ml^−1^ (range, 119–480) after completion of chemotherapy but before definitive surgery (Figure 2). This increase was statistically significant, *P*<0.001. However, there was no difference in the mean baseline serum collagen IV concentration (172 ng ml^−1^) of those who achieved pathologic complete response (pCR=no invasive cancer in the breast or lymph nodes after chemotherapy) and those with lesser response (any residual invasive cancer at surgery), 161 ng ml^−1^; *P*=0.90. Similarly, there was no difference in mean collagen IV concentrations after chemotherapy in the two different response groups (206 ng ml^−1^ each). Post-chemotherapy collagen IV levels were also similar regardless of the type of treatment that was given, chemotherapy alone (181 ng ml^−1^) *vs* chemotherapy plus trastuzumab (191 ng ml), *P*=0.37.

## Discussion

To our knowledge, this is the first study that examined serum collagen IV levels in early stage breast cancer patients and in healthy women. We found that patients with stage II–III breast cancer have significantly higher levels of collagen IV in their blood than healthy women. These results are concordant with previous studies in others type of cancers. Hong *et al* (1995) reported higher concentrations of serum collagen IV in liver carcinoma and in patients with metastatic colon cancer compared to healthy subjects. Also, in colorectal cancer, higher collagen IV levels were detected in patients with liver metastasis compared to those without metastasis (Ambiru *et al*, 1995). Increased levels of this protein in the blood of cancer patients may be an indicator of high level of angiogenesis or may be because of increased degradation due to the invasion process (Hewitt *et al*, 1992). High degree of individual variation including up to fivefold differences in serum collagen IV concentration among cancer patients suggests that this molecule may be worth studying as a potential marker of angiogenic activity.

Our study has several limitations; the sample size is small and does not allow sufficiently powered subset analysis. The median age of the cancer patients was significantly older than the median age of the volunteers. This may introduce a bias and the differences that we report between cancer cases and controls may be due to age. However, we also compared collagen IV levels between younger and older cancer patients and did not detect a significant difference by age. We were not able to examine the impact of timing of the diagnostic core needle biopsy and serum collagen levels. One may hypothesize that the small invasive procedure could on its own lead to increased collagen IV levels due to tissue injury. However, the time between the diagnostic biopsy and collection of serum always exceeded 1 week which makes this hypothesis somewhat less likely. The samples were stored uniformly and spanned a similar collection and storage period; therefore sample handling or differences in storage length are unlikely to contribute to the observed difference in collagen IV levels. We attempted to correlate serum levels with collagen IV expression and angiogenesis in the primary tumour at the time of diagnosis but could only retrieve six of the 51 tumour specimens. This analysis therefore was underpowered to detect any but the most dramatic association. To conclude, we observed a difference in serum collagen IV levels between healthy women and breast cancer patients; however, the small sample size and lack of inclusion of benign breast disease (e.g. *in situ* cancers, adenomas, atypical proliferative lesions) preclude us to define the sensitivity or specificity and positive or negative predictive values of this serum marker. Thus our cohort is too small to establish the use of collagen IV as a biomarker and it is not possible to determine the sensitivity or specificity of collagen IV from this study.

In this study, collagen IV levels increased significantly during preoperative chemotherapy. Similar increase in serum collagen IV was also reported in colon cancer that persisted for several months after treatment (Kudo *et al*, 2002). Why collagen IV levels increased in response to cytotoxic therapy is unclear. It may be due to vascular membrane destruction by chemotherapy or increase in angiogenic activity in response to cellular stress in the cancer induced by chemotherapy. It is intriguing that the post-chemotherapy increase appeared steeper in patients with residual disease (*P*=0.002) compared to those who achieved complete eradication of the cancer (pCR). This suggests that tumour-related factors may regulate the release of collagen IV into the circulation. We did not measure other potential markers of angiogenesis or tissue remodeling during this study. Collagen IV may serve as a potential marker of tumour angiogenesis and further studies may be warranted to examine if patients with higher serum collagen IV concentrations are more susceptible to angiogenesis inhibitors and if changes in collagen IV levels could be used to monitor treatment effect.

## Figures and Tables

**Table 1:** Patient characteristics

**Characteristics**	**Patients *n*=51 (100%)**
*Median age at diagnosis*	52 (range, 21–70)
<50 years	20 (39%)
⩾50 years	31 (61%)
	
*Tumour size*	
T1	10 (19.6)
T2	29 (56.9)
T3	9 (17.6)
T4	1 (2)
NA	2 (3.9)
	
*Clinical nodal status at diagnosis*	
Positive	26 (51)
Negative	23 (45.1)
Unknown	2 (3.9)
	
*Oestrogen receptor status*	
Positive	28 (54.9)
Negative	21 (41.2)
Unknown	2 (3.9)
	
*Progesterone receptor status*	
Positive	20 (39.2)
Negative	29 (56.9)
Unknown	2 (3.9)
	
*HER-2 status*	
Positive	40
Negative	8
Unknown	3
	
*Type of preoperative chemotherapy*	
H+T/FAC	29
T/FEC	13
None	9
